# Evaluation the Effect of Amine Type on the Non-isothermally Derived Activation Energy for the Interaction of 3 Antidepressant Drugs with Lactose

**DOI:** 10.15171/apb.2019.033

**Published:** 2019-06-01

**Authors:** Faranak Ghaderi, Mahboob Nemati, Mohammad Reza siahi-shadbad, Hadi valizadeh, Farnaz Monajjemzadeh

**Affiliations:** ^1^Department of Pharmaceutical and Food Control, School of Pharmacy, Urmia University of Medical Sciences, Urmia, Iran.; ^2^Food and Drug Safety Research Center, Tabriz University of Medical Sciences, Tabriz, Iran.; ^3^Department of Pharmaceutical and Food Control, Faculty of Pharmacy, Tabriz University of Medical Sciences, Tabriz, Iran.; ^4^Pharmaceutical Analysis Research Center, Tabriz University of Medical Sciences, Tabriz, Iran.; ^5^Department of Pharmaceutics, Faculty of Pharmacy, Tabriz University of Medical Sciences, Tabriz, Iran.

**Keywords:** Interaction, DSC, Amine type, Lactose

## Abstract

***Purpose:*** Evaluation of drug-excipients compatibility is an important stage during preformulation studies. In the present research, differential scanning calorimetry (DSC) at different heating rates (2.5, 10, 15°C/min) was applied for the kinetic evaluation of fluvoxamine (FLM), sertraline (SER) and doxepin (DOX) binary mixtures with lactose.

***Methods:*** Solid state kinetic parameters of the mixtures were calculated using two different thermal methods including ASTM E698 and Starink and the effect of amine type (pKa value) was investigated based on the calculated activation energies.

***Results:*** Based on obtained results mean activation energy calculated for FLM, SER and DOX with lactose using ASTM E698 and Starink methods are equal to 335.23, 132.02 and 270.99 kJ/ mol respectively.

***Conclusion:*** Results showed that the probability of drug-lactose interaction is higher in the SERlactose mixture in comparison with other two antidepressant drugs which is consistent with their pKa values.

## Introduction

Preformulation studies supporting development of safe and high quality dosage forms. Physical and chemical properties of drug agents are considered in select of formulation ingredients. Compatibility of dosage form components and kinetic study are main objectives of preformulation studies in order to develop the stable, safe, efficient and high quality dosage forms. Drug-excipient interaction is one of the most common incompatibility reported. Excipients are added to formulations to facilitate manufacture, absorption, stability and administration. Lactose is a reducing disaccharide and one of the most widely used filler in oral formulations.

The reaction of lactose with pharmaceutical active ingredients have been reported in some cases such as acyclovir, gabapentin, metoclopramide, baclofen, methyldopa and fluoxetine.^1-7^ Various physicochemical methods were used to study the interaction between amine containing drugs and reducing carbohydrates such as differential scanning calorimetry (DSC), FTIR, MS, NMR and HPLC.

Multiple scanning method using DSC at different heating rates and isoconversional calculation procedures are fast and easy alternatives to the conventional method, in order to calculate solid-state kinetic parameters.^8-10^

To the best of our knowledge, there is no relevant study evaluating the effect of amine type on the non-isothermally derived activation energy for the interaction of pharmaceutical active agents with lactose.

**Figure 1:**
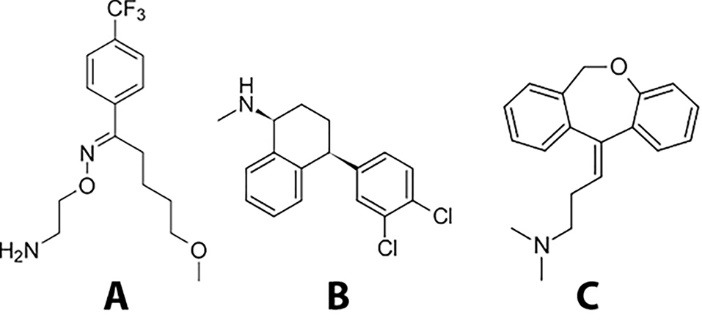
Chemical structure of (A) FLM, (B) SER and (C) DOX.

In the present study thermokinetic and thermodynamic parameters of the interaction of FLM (fluvoxamine), SER (sertraline) and DOX (doxepin) as model drugs and containing primary, secondary and tertiary amines with lactose were calculated based on DSC non-isothermal kinetic methods. Chemical structures of the mentioned drugs are shown in Figure 1.

## Materials and Methods

### Material

DOX, SER and FLM were purchased from Dipharma Francis Pharmaceutical Co. (Baranzate, Italy), Novin Kavosh Mamatir Co. (Tehran, Iran) and TEMAD Co. (Karaj, Iran) respectively. Anhydrous lactose was provided from DMV Chemical Co. (Veghal, Netherlands).

### Differential scanning calorimetry

A DSC-60, Shimadzu differential scanning calorimeter (Kyoto, Japan), with TA-60 software (version 1.51) was used for thermal analysis of drugs and excipient binary mixtures. Binary samples (10 g) were prepared (1:1 mass ratio of drugs and excipient) and uniform mixing was achieved by tumbling method. Five milligrams of each sample was weighed and compressed in the DSC aluminum pan, and pressed using a cap. Then, it was scanned in the temperature range of 25–300°C, with different heating rates (2.5, 10 and 15°C/min).

## Results and Discussion

**Figure 2:**
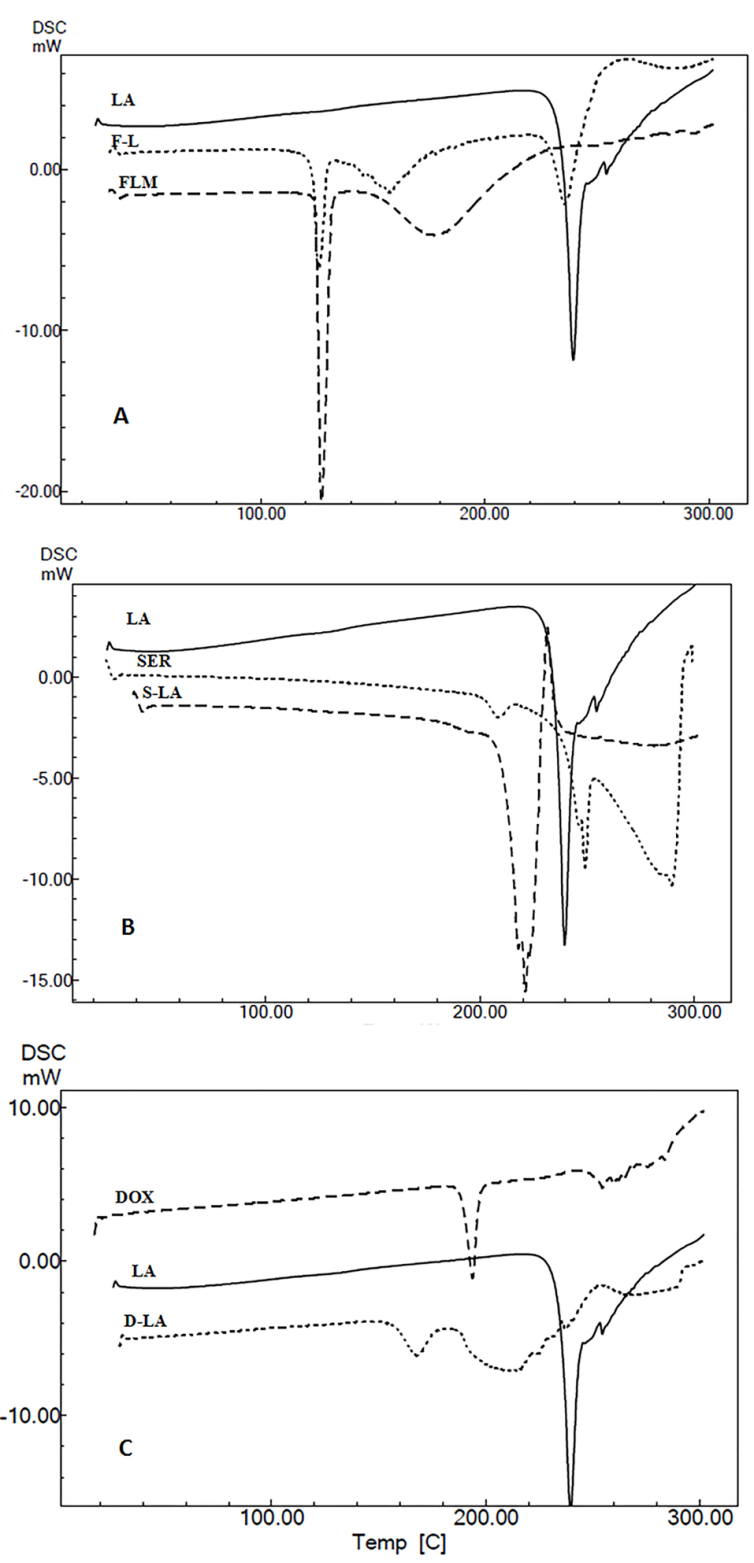
Selected DSC thermograms of (A) FLM, lactose and FLM- lactose 1:1 W/W binary mixture^10^(B) SER, lactose and SER- lactose 1:1 W/W binary mixture^11^ (C) DOX, lactose and DOX- lactose 1:1 W/W binary mixture^12^ (β=10) [Presented with permission].

Selected DSC curves of drug, excipient and drug-excipient mixture are shown in Figure 2. Thermal behavior of pure drug, excipient and their binary mixture were compared in the DSC curves.

According to Figure 2A, B and C the endothermic peak of pure FLM, SER, DOX and pure anhydrous lactose was appeared at127.2, 248.9, 193.8 and 239.1°C respectively which can be related to melting phenomena.^13^

In FLM-lactose mixture no peak has been added or nor is removed. Thus simple DSC method is incapable to follow the possible interaction and it may ignore the drug- excipient incompatibility (Figure 2A).

Disappearance of SER melting peak and also formation a new endothermic peak at 215.7°C in drug-excipient mixture can only due to the interaction between the mixture components under non isothermal DSC conditions (Figure 2B).

Also disappearance of melting peak of DOX and generation a new endothermic peak at 167.9°C in the DOX–lactose binary mixture, may be related to drug- excipient interaction (Figure 2C).

**Figure 3:**
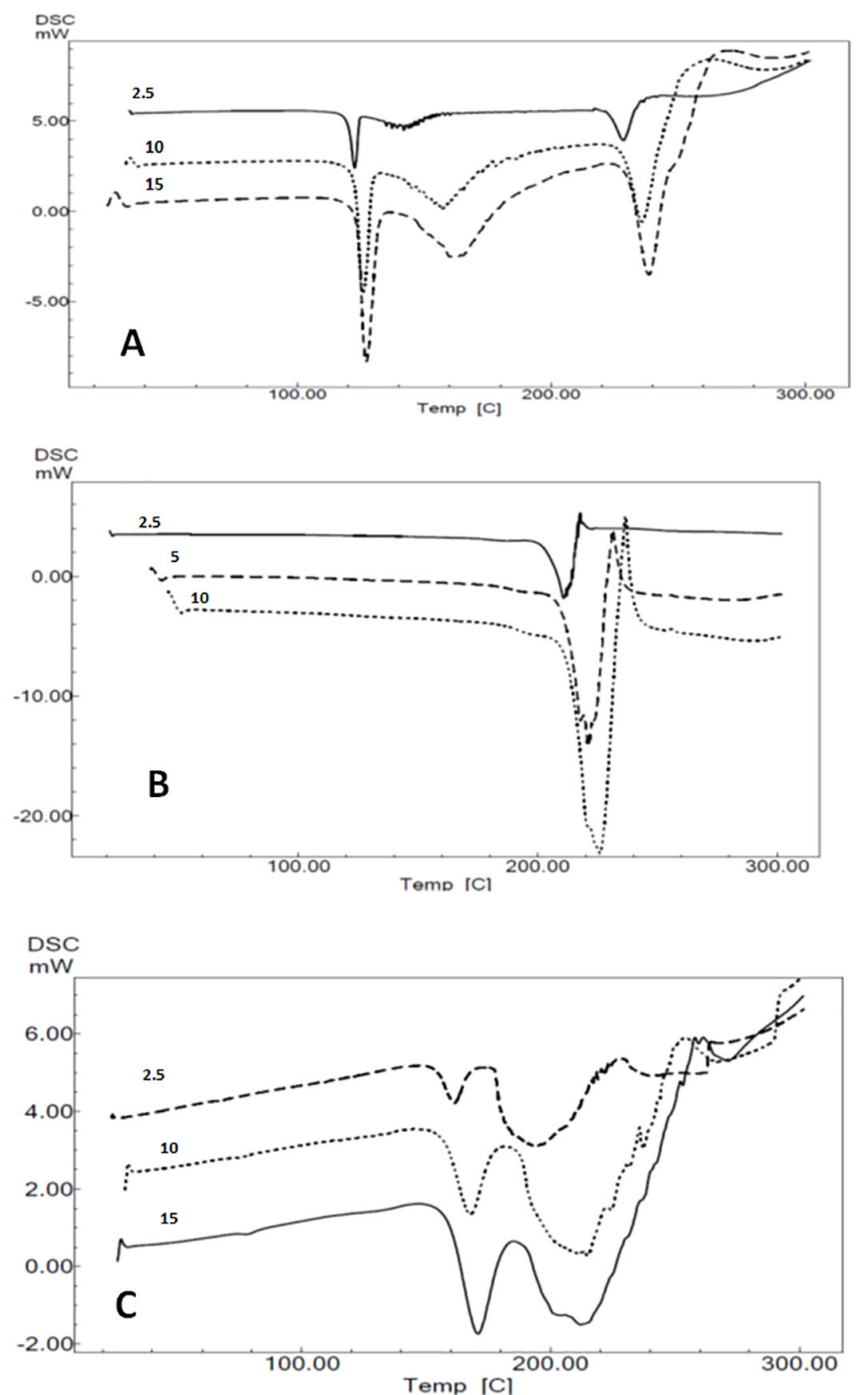
Selected DSC thermograms of (A) FLM-lactose^10^ (B) SER-lactose^11^ (C) DOX-lactose 1:1 binary mixtures at different heating rates (β=2.5, 10, 15) [Presented with permission].

Multiple scan method using isoconversional calculation procedures have been recently used by many researchers in assessment of pharmaceutical product stability and also kinetic study.^8,11,12^ While increasing heating rate DSC thermograms were shifted to higher temperature and this issue was used to calculation of kinetic parameters.^14,15^ DSC thermograms of FLM-lactose, SER-lactose and DOX-lactose 1:1 binary mixtures at different heating rates are presented in Figure 3.

**Figure 4:**
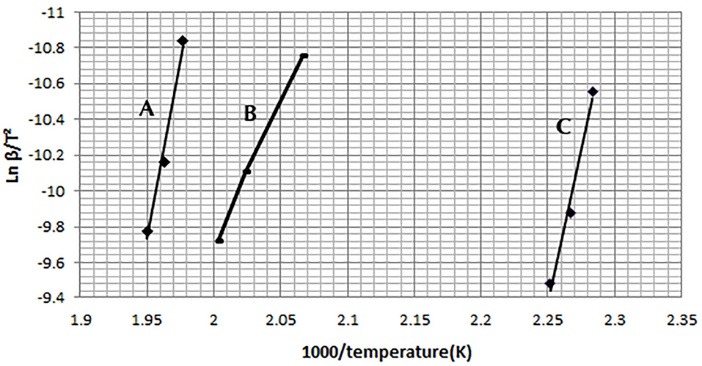
ASTM’s plot for (A) FLM-lactose (B) SER-lactose (C) DOX-lactose mixture with 1:1 mass ratio at different heating rates (β=2.5, β=10, β=15).

In the present study, two recognized methods including ASTM E698 and Starink were used for the assessment of the kinetic parameters of drug-excipient interaction.^16,17^ According to ASTM E698 method, Ln (β/T_m_^2^) is plotted against 1/T_m_, where T_m_ is the maximum peak temperatures of DSC curves obtained at different heating rates (β).^17,18^ The values of the activation energy (*E*) were calculated from the slopes of the straight lines in Figure 4 from ASTM E698 plot and are shown in Table 1.

**Table 1:** Kinetic parameters for the FLM-lactose, SER-lactose and DOX-lactose interactions obtained by ASTM and Starink Methods

**Drug -lactose**	**Kinetic method**	**Activation energy (KJ.mol** ^−1^ )	**Arrhenius Factor LogA(s** ^−1^ )	**ΔG (kJmol** ^−1^ )	**ΔH (kJmol** ^−1^ )	**ΔS (Jmol** ^−1^ **K** ^−1^ )	**Rate constant** **k (s** ^−1^ )
FLM	ASTM	335.06	32.78	142.45	330.83	0.369	8.96
	Starink	335.40	32.81	142.45	331.17	0.370	8.97
SER	ASTM	131.85	11.96	141.82	127.75	-0.028	7.20
	Starink	132.19	11.99	141.81	128.8	-0.027	7.21
DOX	ASTM	270.85	30.52	122.59	267.18	0.327	8.92
	Starink	271.14	30.55	122.58	267.47	0.328	8.92

Also, as suggested by ASTM E698, the values of frequency factor could be calculated from the following equation (Table 1):

Eq. (1) A=β(ERT2)e(ERT)

In which, *T* is the temperature (K), *β* is the heating rate (°C/min), *E* is the activation energy (kJ/mol), *A* is the frequency factor and R is the gas constant.

**Figure 5:**
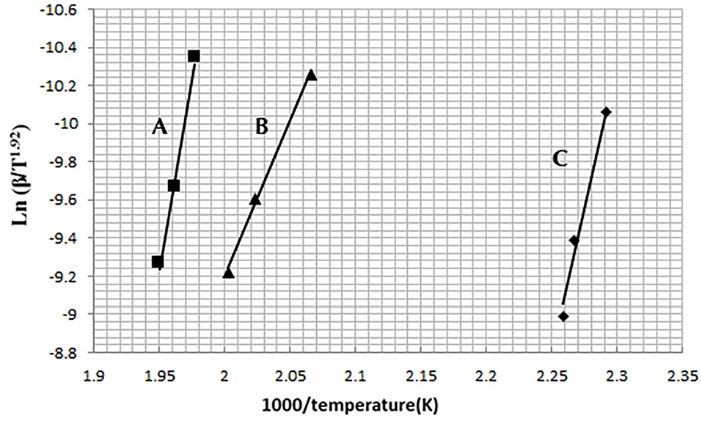
Starink’s plot for (A) FLM-lactose (B) SER-lactose (C) DOX-lactose mixture with 1:1 mass ratio at different heating rates (β=2.5, β=10, β=15).

In the Starink method, Activation energy (*E*) was obtained from the slopes of the straight lines of the Ln (β/T_m_^1.92^) plot vs. (1/T_m_) presented in Figure 5. In which Tm (K) is maximum peak temperature of DSC thermogram at different heating rates (β).^16,19^ The frequency factor (A) was also calculated from Eq. (1).

The resulting thermokinetic parameters for drugs-lactose interaction for both methods are also presented in Table 1.

The calculated kinetic parameters were used to assess the thermodynamic parameters of activation including change in entropy (ΔS), enthalpy (ΔH), and free energy (ΔG) related to the activation using the following equations.

Eq. (2) $$Ae^{\frac{E}{RT}}=ve^{\frac{\Delta G}{RT}}$$

ΔH = E – RT Eq. (3)

ΔG = ΔH – TΔS Eq. (4)

In Eq. (2), υ=k_B_T**/** h (where *k*_B_ is Boltzmann constant and *h* is Plank constant). The obtained thermodynamic parameters of activation for this interaction are also presented in Table 1.

The values of reaction rate constants (k) for interaction of drugs-lactose mixture were calculated at the room temperature according to the Eq.5^20^ and the aforementioned values for activation energies and frequency factors for these mixtures were presented in Table 1.

LogK=LogA−E2.3RT

As shown in Table 1 the mean activation energy calculated for FLM, SER and DOX(containing primary, secondary and tertiary amine) mixtures with lactose using ASTM and starink methods are equal to 335.23, 132.02 and 270.99 KJ/mol respectively.

pK_a_ values of FLM , SER and DOX are 8.7, 9.48 and 9 respectively and these values are in compliance with resulted activation energy values.^13^

According to chemistry, the amine type can influence the reaction of these pharmaceutical agents with lactose as an electronucleophilic reaction. Thus it was supposed the tertiary amine be more reactive than the secondary and primary amine functionality in the assumed interaction. But the results of Table 1 indicates SER (secondary amine) as the most reactive molecule compared with DOX (tertiary) and FLM (primary amine). In the interpretation of the results, it can be said that SER acts as a stronger base than others and SER-lactose interaction has the lowest activation energy value. In addition the amine functionality substituents of SER molecule are more electron donor than DOX and FLM. Besides in SER the adjacent electron donor groups are located very close to the amine functionality. So it can be concluded that SER is most reactive than two others.

Likewise, despite that FLM has primary amine but due to the lowest pK_a_ value than the two others and less electron donor substituents and steric hindrance effects the activation energy of FLM - lactose activation energy is very high and thus the molecule is less liable to interaction. The results indicate that in pharmaceutical molecules there are more other determining factor which should be considered along with the pK_a_ values and DSC kinetic evaluation provides an easy way to calculate kinetic parameters in solid state mixtures.

## Conclusion

Thermokinetic and thermodynamic parameters corresponding to drug – excipient interaction in some antidepressant drugs containing amine group were calculated using DSC curves obtained by multiple scan method at various heating rates. Two well-known kinetic methods including ASTM and Starink were used to calculate the activation energies and Arrhenius factor for the drug-excipient interactions and results was applied for interpretation of the amine type effect on the interaction. Based on the obtained results the probability of this reaction is higher in the SER-lactose mixture which contains secondary amine type. The results indicate that in pharmaceutical molecules there are more other determining factor which should be considered along with the pk_a_ values and DSC kinetic evaluation provides an easy way to calculate kinetic parameters in solid state mixtures and to make a good prediction available for industrial pharmacists.

## Ethical Issues

Not applicable.

## Conflict of Interest

The authors declare no financial or other conflict of interest.

## Acknowledgments

This work is a part of a thesis by Faranak Ghaderi submitted for PhD degree (No. 91) and is supported by faculty of Pharmacy, Tabriz University of Medical Sciences.
